# Effects of Circular RNA of Chicken Growth Hormone Receptor Gene on Cell Proliferation

**DOI:** 10.3389/fgene.2021.598575

**Published:** 2021-02-11

**Authors:** Haidong Xu, Qiying Leng, Jiahui Zheng, Patricia Adu-Asiamah, Shudai Lin, Ting Li, Zhang Wang, Lilong An, Zhuihui Zhao, Li Zhang

**Affiliations:** ^1^College of Coastal Agricultural Sciences, Guangdong Ocean University, Zhanjiang, China; ^2^Guangdong Provincial Key Laboratory of Agro-Animal Genomics and Molecular Breeding, Key Laboratory of Chicken Genetics, Breeding and Reproduction, Ministry of Agriculture, South China Agricultural University, Guangzhou, China

**Keywords:** chicken, growth hormone receptor, circular RNA, cell proliferation, linear transcript

## Abstract

Animal growth and development are regulated by neural and endocrine growth axes, in which cell proliferation plays key roles. Recently, many research showed that circular RNAs were involved in hepatocyte and myoblast proliferation. Previously, we identified a circular RNA derived from the chicken *GHR* gene, named circGHR. However, the function of circGHR is unclear. The objective of this study was to investigate circGHR expression pattern and its roles in cell proliferation. Results indicated that circGHR was a closed-loop structure molecule, and it was richer in the nucleus of hepatocytes and myoblast. Real-time PCR showed that circGHR was increased from E13 to the 7th week in the liver but decreased in the thigh and breast muscle. The CCK-8 assay displayed that circGHR promoted cell proliferation. Simultaneously, the biomarker genes *PCNA*, *CCND1*, and *CDK2* and the linear transcripts *GHR* and *GHBP* were upregulated when circGHR was overexpressed. Altogether, these data exhibited that circGHR could promote cell proliferation possibly by regulating *GHR* mRNA and *GHBP* expression.

## Introduction

Animal growth and development are regulated by the neural and endocrine growth axes, including the hypothalamus, pituitary, growth hormone (GH), and target organs (Picard et al., 2002). GH is transported to the target organ by growth hormone binding protein (GHBP) in body fluid (Picard et al., 2002). Then, it can combine with the growth hormone receptor (GHR) to activate the intracellular insulin-like growth factor (IGF) pathway and involve in cell proliferation and differentiation (Sperling, 2016).

Cell proliferation is the starting point for tissue and organ development. Hepatocyte proliferation is the primary biological process in liver development during the early embryonic stage (Hamada et al., 1995). Also, muscle development begins with myoblast proliferation, which depends on the increased number of muscle fibers (Picard et al., 2002; Welle et al., 2007). Once the growth phase is completed, the fiber numbers do not increase, but the fiber volume increases (Buckingham et al., 2003). Recently, many researchers identified circular RNAs (circRNAs) regulating hepatocyte and myoblast proliferation, such as circMark14 (Li L. et al., 2017) and circFUT10 (Li H. et al., 2017).

CircRNAs, a class of endogenous molecules with covalent-formed closed-loop structure and abundant in eukaryotic organisms (Conn et al., 2015), functioned in the skeletal muscle development of animals by regulating gene expression at multiple levels. Most researchers exhibited that circRNAs functioned by multiple mechanisms, such as miRNA sponges, translation, and regulating their parental genes (Patricia et al., 2020). Specifically, bovine circFUT10 regulated myoblast differentiation and cell survival by directly binding to miR-133a and inhibiting miR-133a activity (Li H. et al., 2017), while human and mouse circ-ZNF609 consisted of direct reading scaffolds, which can translate into protein and play roles in skeletal muscle disorders (Legnini et al., 2017). Another interesting circRNA was circMbl. Findings confirmed that muscleblind (MBL/MBNL1) could bind to its pre-mRNA and encouraged circMbl production, which may be associated with myotonic dystrophy initiation and progression (Ashwal-Fluss et al., 2014).

Previously, we identified a circRNA divided from the chicken *GHR* gene, named circGHR (Zhang et al., 2019). Yet, the function and regulation mechanisms of circGHR are uncertain. This study mainly analyzed its expression patterns and its effects on cell proliferation (the summary technical pipeline is in Supplementary Figure 1). We found that chicken circGHR could promote cell proliferation, possibly by regulating *GHR* mRNA and *GHBP* expression.

## Materials and Methods

### Ethics Statement

Animals involved in this research were humanely sacrificed as necessary to ameliorate suffering. The study was approved by the Animal Care Committee of Guangdong Ocean University (Zhangjiang, China).

### Experimental Animal and Sample Collection

Body tissues including breast muscle, thigh muscle, liver, heart, and small intestine were collected from four male Huaixiang chickens (a local Chinese breed) at every age from embryonic 13 (E13) to 7 weeks (7W) (E13, E16, E19, 1D, 1W, 2W, 3W, 4W, 5W, 6W, and 7W). Besides, the tissue samples were snap-frozen in liquid nitrogen and stored in a freezer at −80°C until analyzed. All the fertile chicken eggs were obtained from the HuaiXiang Chicken Breeding Farm (Yingfu Company, Xinyi City, Guangdong Province). They were incubated in an automatic incubator (Baihui, Shandong Province) at 37.8°C, with 50–60% humidity. Chicken myoblast and hepatocytes were individually isolated at E11 and E16 during incubation. The broilers were fed with the normal basal diet, including 11.51% metabolic energy, 15.23% crude protein, 3.10% calcium, 0.45% available phosphorus, and a few non-essential amino acids.

### Identification of Chicken CircGHR Molecular Characteristics

In our previous study, we characterized circGHR by RNase R treatment and sequencing (Zhang et al., 2019). Here, we compared the reverse transcriptional efficiency with two kinds of primers. Firstly, the cDNA was synthesized by PrimeScript RT Reagent Kit (TaKaRa, Kyoto, Japan) with random hexamer or oligo(dT)_18_ primers, respectively. Then, qRT-PCR was conducted to compare the relative level between the two groups. The *GAPDH* gene was used as an internal control. Additionally, the amplification products were detected using agarose gel electrophoresis and Sanger sequencing. The primers used in this experiment are listed in Table 1.

**TABLE 1:** Primers used in this study.

Primer name	Primer sequence (5′ → 3′)	Transcript or Gene ID	Tm/°C	Product/bp	Application
circGHR-F	GTCCCTCAGCTCAACTGC		53.0	115	circGHR quantitative PCR
circGHR-R	ATCTTCGGCATCTGCTGT				
*GHR*-F	AGTCCGATCAAGACAACGTAC	XM_015272680.1	59.4	128	*GHR* mRNA quantitative PCR
*GHR*-R	CTAAGAACCAGGGAAACTCG				
*GHBP*-F	TGATGAAATAGTACTACCTGATCC	DQ138367	56.3	208	*GHBP* quantitative PCR
*GHBP*-R	TAAATATTTCCTCCATACCTCC				
circGHR-V-F	gg*GGTACC*tgaaatatgctatcttacagGTGCTGGGATGGCTGGAGAAGG		70.0	221	Construct circGHR expression vector
circGHR-V-R	cg*GGATCC*tcaagaaaaaatatattcacCATCACTTGCAGAAAGTGAGTCATT				
GHR-luc-F	*tcttacgcgtgctagCCCGGG*CAGTTAGGCAAGTAAATGTATATTGGA	408184	60.0	2,998	Construct GHR promoter reporter vector
GHR-luc-R	*acttagatcgcagatCTCGAG*CTGCTAACCTCCTTCTCTAGGTATGC				
GHBP-luc-F	*tcttacgcgtgctagCCCGGG*CTTAGATTAAAACCTCTGCGAG	408184	60.0	1,430	Construct GHBP promoter reporter vector
GHBP-luc-R	*acttagatcgcagatCTCGAG*GGGAAGGAGGGGATGAGGGA				
*PCNA*-F	CTCTGAGGGCTTCGACACCT	NM_204170.2	58.0	133	*PCNA* quantitative PCR
*PCNA*-R	ATCCGCATTGTCTTCTGCTCT				
*CCND1*-F	AACCCACCTTCCATGATCGC	NM_205381.1	57.8	159	*CCND1* quantitative PCR
*CCND1*-R	CTGTTCTTGGCAGGCTCGTA				
*CDK2*-F	GTACAAGGCCCGGAACAAGG	NM_001199857.1	58.2	168	*CDK2* quantitative PCR
*CDK2-*R	TTCTCCGTGTGGATCACGTC				
*GAPDH*-F	AGGACCAGGTTGTCTCCTGT	NM_204305.1	60.0	153	Internal control in qPCR analysis
*GAPDH*-R	CCATCAAGTCCACAACACGG				
*Sno-U6*-F	CTCGCTTCGGCAGCACA	X07425.1	53.0	94	
*Sno-U6*-R	AACGCTTCACGAATTTGCGT				

*The underlined and italic type is the enzyme site of *Kpn*I and *Bam*HI and the lowercase part is the filling sequence and protecting base for circGHR-V primers. The underlined and italic type is the enzyme site of *Sma*I and *Xho*I and the lowercase part is the homologous sequences with pGL3-basic for GHR/GHBP-luc primers.*

### Cell Culture

Two types of chicken primary cells (myoblast and hepatocyte) and two types of chicken cell lines (LMH and DF-1 cell lines) were cultured for analyzing the roles of circGHR.

Myoblast was isolated from chicken thigh muscles at E11 according to previously described methods (Zhang et al., 2017). Briefly, a differential attachment was performed three times every 40 min after the thigh muscles were cut into pieces. During the third time, the adherent cells were collected and directly grown on a plastic plate. The cells were cultured in growth medium (GM) with DMEM-F12 (Gibco, New York, United States), 15% fetal bovine serum (Gibco, New York, United States), and 1% penicillin/streptomycin (Gibco, New York, United States) in 5% CO_2_ incubator at 37°C.

Hepatocytes were isolated from the chicken liver at E16 according to previously described methods (Wang et al., 2019). Briefly, the liver was washed with phosphate buffer saline (PBS) three times to remove the impurities. It was then cut into pieces and digested using collagenase type V at 34°C for 7 min. The cellular suspension was filtered through 200 and 500 mesh sieves, respectively, and washed with PBS three times at 1,000 rpm for 5 min. The obtained cells were isolated by non-continuous density Percoll gradient centrifugation (Sigma, MO, United States) and diluted with Williams’ E uncomplete medium (Sigma, no fetal bovine serum) to the concentration of 1 × 10^6^ cells/ml, followed by plating into 12-well dishes. After incubation in 5% CO_2_ incubator at 37°C for 4 h, the medium was changed with Williams’ E complete medium containing 10% fetal bovine serum and 1% penicillin/streptomycin solution (Gibco, New York, United States) until treatment.

Chicken LMH and DF-1 cell lines, provided by South China Agricultural University, were cultured with DMEM containing 10% fetal bovine serum and 1% penicillin/streptomycin solution (Gibco, New York, United States) in 5% CO_2_ incubator at 37°C.

### Nucleus and Cytoplasm Separation

To analyze the location of circGHR in the cells, the nucleus and cytoplasm RNA were extracted from three wells of chicken myoblast or hepatocytes by PARIS^TM^ Kit Protein and RNA Isolation System (Invitrogen, Carlsbad, CA, United States) according to the manufacturer’s instructions. The PARIS method was based on differential lysis of plasma and nuclear membranes by non-ionic detergents. Briefly, cells were first separated into nuclear and cytoplasmic fractions, and then RNA was isolated. The cell pellet was suspended in a buffer for RNA purification and then centrifuged twice at 4°C for 5 min. The supernatant was the cytoplasmic fraction, and the pellet was the nucleus fraction. RNAs were extracted from both fractions using TRIzol (Mange, Guangzhou, China). The relative levels of circGHR between the nucleus and cytoplasm were compared by qRT-PCR. Both *sno-U6* and *GAPDH* were used as control.

### Bioinformatics Analysis

The transcription start sites and promoter regions of *GHR* and *GHBP* were analyzed by referencing the thesis of Lau and Joanna (2005). The conserved transcription factor binding sites in the vertebrate database were predicted using the JASPAR website tool^1^ (Ovcharenko et al., 2005).

### CircGHR Plasmid Construction and Cell Transfection

For the circGHR circular overexpression vector (pCD2.1-circGHR), the full length of circGHR was amplified by PCR with forward and reverse primers, including *Kpn*I and *Bam*HI restriction enzyme sites at 5′-ends, respectively (Table 1). The PCR was performed with a 20-μl reaction mixture containing 1 μl of cDNA transcribed from chicken liver RNA, 10 μl of TaKaRa Taq Version 2.0 plus dye (TaKaRa, Kyoto, Japan), 1 μl each of the forward and reverse primers (10 μM), and double-distilled water. The PCR reaction procedure was as follows: 94°C 2 min, 40 cycles at 94°C 30 s, 57°C 30 s, and 72°C 30 s, followed by 72°C for 5 min. The PCR products were digested with *Kpn*I and *Bam*HI restriction enzymes. The purified fragment was ligated into pCD2.1-ciR(+) plasmid (with green fluorescent protein tag, Geneseed Company, Guangzhou, China) to obtain the pCD2.1-circGHR recombinant plasmid. pCD2.1-circGHR overexpression experiment was carried out in a 12-well plate by transient transfecting pCD2.1-circGHR into the cell using Lipofectamine 3000 (Invitrogen, Carlsbad, CA, United States) according to the manufacturer’s protocol.

Genomic DNA was isolated from chicken liver samples (*n* = 3) using the phenol–chloroform method (Green and Sambrook, 2014) and stored at −20°C. For the construction of *GHR* and *GHBP* promoter-luciferase reporter vector, chicken *GHR* and *GHBP* promoter fragments were amplified with primers GHR/GHBP-Luc (Table 1) using genomic DNA. Subsequently, the amplified *GHR* and *GHBP* promoter fragments were forwardly inserted into the promoter-less luciferase reporter vector pGL3-basic (Promega, United States) between *Sma*I and *Xho*I sites and named pGL3-GHR(−2,730/ +226) and pGL3-GHBP(−1,322/ +66), respectively (Lau and Joanna, 2005). DF-1 cells were transiently co-transfected with either luciferase report vector [pGL3-GHR(−2,730/ +226) or pGL3-GHBP(−1,322/ +66)] and pCD2.1-circGHR or pCD2.1-ciR plus pRL-TK Renilla luciferase vector as above.

All primers were synthesized by Sangon Biotech (Shanghai, Chain), and all plasmids were confirmed by DNA sequencing (Sangon, Shanghai, Chain).

### Cell Counting Kit-8 Assay

The Cell Counting Kit-8 (CCK-8) assay was used to examine cell proliferation. Briefly, the cells were plated into 96-well culture plates at a density of 1 × 10^4^ cells/well in 100 μl of culture medium per well. In the beginning, the CCK-8 reagent was used to select the uniform well for transfection, and each group had 12 independent replicates. After transfecting pCD2.1-circGHR recombinant plasmid or control pCD2.1-ciR(+) plasmid for 12 h, 10 μl of CCK-8 reagent (Dojindo Laboratories, Kumamoto, Japan) was added to each well and incubated at 37°C for 2 h. Each sample’s absorbance was detected at 450 nm using a microplate reader (Thermo Fisher, MA, United States). Cell proliferation was then monitored every 12 h according to the manufacturer’s protocol.

### Dual-Luciferase Reporter Assay

Briefly, the DF-1 cells (2 × 10^5^ cells/well) were seeded in a 24-well plate and cultured in the medium. After reaching 70–80% confluence, the cells were washed with PBS, and transient transfection was performed using Lipofectamine 3000 (Invitrogen, United States). Dual-luciferase reporter assays were performed 48 h post-transfection using the dual-luciferase reporter assay system (Promega, United States) according to the manufacturer’s instructions, in which Firefly luciferase (*Fluc*) activity was normalized to Renilla luciferase (*Rluc*) activity.

### RNA Extraction and Quantitative Real-Time PCR

Following the manufacturer’s protocol, total RNA was extracted from the cells by TRIzol reagent (Mange, Guangzhou, China). The RNA integration and concentration were detected using electrophoresis at 1.5% agarose gel and Nanodrop 2000 spectrophotometry. cDNA was synthesized with PrimeScript RT Reagent Kit (TaKaRa, Toyoto, Japan). Besides, cDNA was diluted four times with RNase-free water and stored at −20°C. Real-time PCR was conducted in a Bio-Rad CFX96 Real-Time Detection System (Bio-Rad, Hercules, CA, United States) using TransStart Green qPCR SuperMix (Transgen Co., Ltd., Beijing, China). The primers of circGHR, *GHR* mRNA, and *GHBP* and biomarker genes, like *PCNA*, *CCND1*, and *CDK2* (Fukami-Kobayashi and Mitsui, 1999), are listed in Table 1. The expression was normalized with *GAPDH*. We performed qRT-PCR with 20 μl mixture containing 1 μl of cDNA, 10 μl of 2× TransStart Green qPCR SuperMix, 0.5 μl each of the forward and reverse primers (10 μM), and double-distilled water. The qRT-PCR reaction procedure is as follows: 95°C for 30 s, 40 cycles at 95°C for 5 s, annealing for 30 s, 72°C for 30 s, followed by 72°C for 5 min. The expressed gene was quantified using the comparative threshold cycle (2^–ΔΔ^ ^*Ct*^) methods.

### Statistical Analysis

Each experiment was repeated three times and all the data were expressed as the mean ± SE and processed using the statistical software SAS 9.1.3 (SAS Institute Inc., NC, United States). Unpaired Student’s *t* test was used for *P* value calculations. GraphPad Prism 8.3 was used for the creation of boxplot figures. A single asterisk (^∗^) was considered significant (*P* < 0.05), whereas double asterisks (^∗∗^) (*P* < 0.01) were considered extremely significant between groups.

## Results

### Chicken CircGHR Characteristics

**FIGURE 1:**
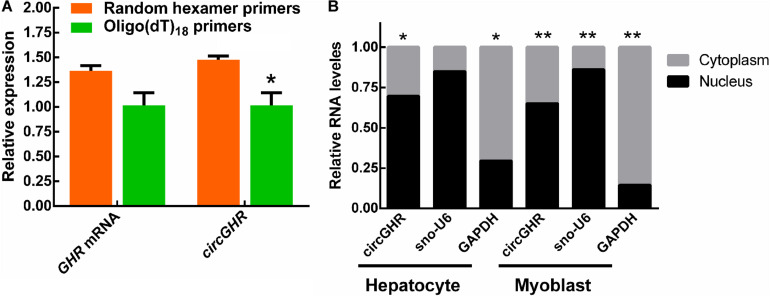
CircGHR characteristic confirmation and its expression pattern in chicken cells. **(A)** The relative expression of *GHR* mRNA and circGHR after specific RT-PCR using oligo(dT)_18_ or random hexamer primers. Fold change was relative to the expression of the random hexamer primers group. **(B)** CircGHR subcellular localization in chicken hepatocytes and myoblast. The RNA relative levels in the nucleus and cytoplasm were calculated by the 2^– *Ct*^ methods. Fold change was relative to the expression of the nucleus. All data are representative of three independent experiments and are shown as the mean ± SEM. *P < 0.05 and **P < 0.01.

In our previous study, we found that the chicken *GHR* gene may possess a circular transcript (circGHR, GenBank accession number is MW145198) transcribed from its 5′ UTR, exon1, and exon2. The circGHR was resistant to RNase R treatment, confirming its circularized characteristics (Zhang et al., 2019). In this study, we further compared its reverse transcriptional efficiency with different primers. The results showed that random primers had higher efficiency than the oligo(dT)_18_ primers, further demonstrating that circGHR was a circular molecule without poly(A) sequences. Meanwhile, the linear transcript *GHR* mRNA had no significant differences in both groups (Figure 1A). In the following experiment, all the RNA was transcribed with oligo(dT)_18_ and random mixture primers. To understand the possible roles of circGHR in hepatocytes and myoblast, we analyzed the location of circGHR, and the results showed that circGHR was richer in the nucleus than in the cytoplasm in both hepatocytes and myoblast (Figure 1B).

### Expression Patterns of CircGHR in Chicken Growth

**FIGURE 2:**
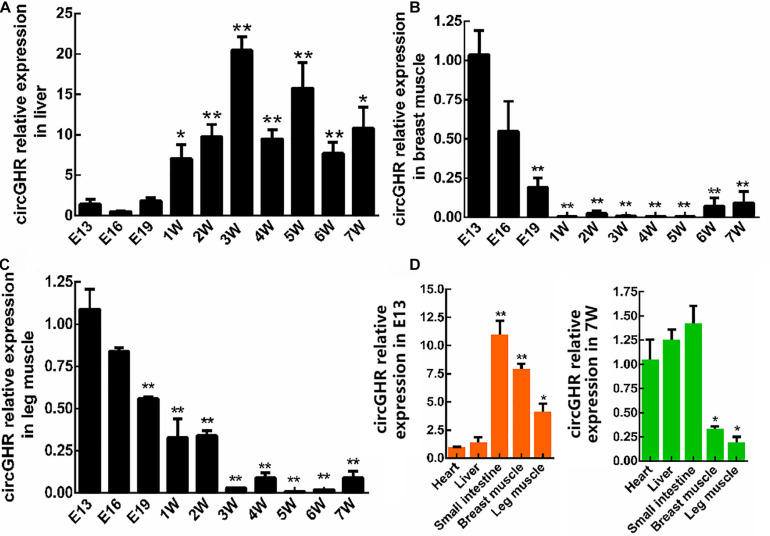
CircGHR relative expression in tissue at different times. **(A–C)** CircGHR time expression profile in the liver, thigh, and breast muscle. Fold change was relative to the expression of E13. **(D)** CircGHR tissue expression profile in E13 and 7W. The RNA relative levels were calculated by the 2^– Δ^ ^Δ^ ^*Ct*^ methods. Fold change was relative to the expression of heart tissues. All data are representative of three independent experiments and are shown as the mean ± SEM. *P < 0.05 and **P < 0.01.

To investigate the expression patterns of circGHR, chicken liver, thigh, and breast muscle were collected from the age of E13 to 7W. The qRT-PCR analysis showed that circGHR expressed increased from 3W to 7W in the liver (Figure 2A) and decreased from E13 to 7W of age in the thigh and breast muscle (Figures 2B,C). Comparing different tissues, we found that a higher level of circGHR expression was in the small intestine, breast, and thigh muscle than in the other tissues at the age of E13 (Figure 2D), while in the heart, liver, and small intestine, the level of circGHR expression was higher than in the other tissues at the age of 7W (Figure 2D). These results showed that circGHR was always highly expressed in the small intestine at both E13 and 7W, which inspired us to resolve whether circGHR regulates cell proliferation or is involved in material absorption and tissue development.

### Effects of CircGHR on Cell Proliferation

**FIGURE 3:**
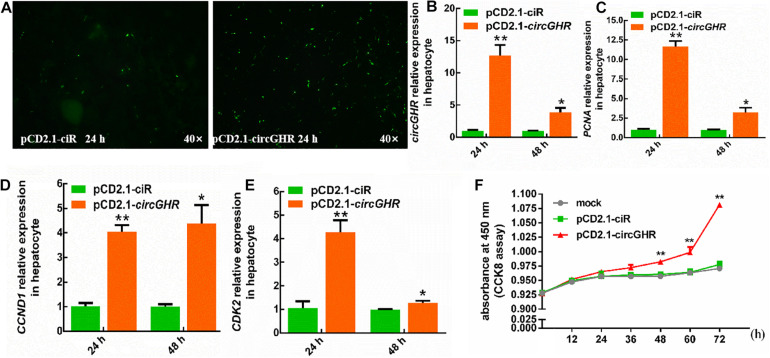
The cell status and the expression profile of the proliferation gene after circGHR overexpression in hepatocytes. **(A)** The status of hepatocytes after transfection vectors 24 h. **(B–E)** The expression profile of circGHR, *PCNA*, *CCND1*, and *CDK2* after circGHR overexpression in hepatocytes. The RNA relative levels were calculated by the 2^– Δ^ ^Δ^ ^*Ct*^ methods. Fold change was relative to the expression of the cells transfected with empty vector pCD2.1-ciR at the corresponding time. **(F)** Hepatocyte growth curves following transfection of pCD2.1-ciR and pCD2.1-circGHR. Fold change was relative to the initial value. All data are representative of three independent experiments and are shown as the mean ± SEM. *P < 0.05 and **P < 0.01.

**FIGURE 4:**
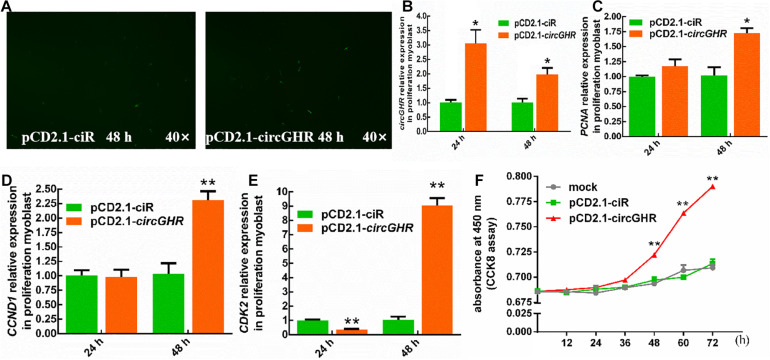
The cell status and the expression profile of the proliferation gene after circGHR overexpression in proliferation phase myoblast. **(A)** The status of myoblast after transfection vectors 48 h. **(B–E)** The expression profile of circGHR, *PCNA*, *CCND1*, and *CDK2* after circGHR overexpression in myoblast. The RNA relative levels were calculated by the 2^– Δ^ ^Δ^ ^*Ct*^ methods. Fold change was relative to the expression of the cells transfected with empty vector pCD2.1-ciR at the corresponding time. **(F)** Myoblast growth curves following the transfection of pCD2.1-ciR and pCD2.1-circGHR. Fold change was relative to the initial value. All data are representative of three independent experiments and are shown as the mean ± SEM. *P < 0.05 and **P < 0.01.

To evaluate the roles of circGHR in cell proliferation, chicken hepatocytes and myoblast were transfected with pCD2.1-ciR or pCD2.1-circGHR, respectively. The expression of proliferation marker genes (*PCNA*, *CCND1*, and *CDK2*) was detected. Quantitative RT-PCR showed that overexpression of circGHR in hepatocytes and myoblast (Figures 3A,B, 4A,B) significantly promoted *PCNA*, *CCND1*, and *CDK2* expression (Figures 3C–E, 4C–E). Consistent with real-time PCR results, the CCK-8 assay results showed that the absorbance of hepatocytes and myoblast transfected with pCD-2.1-circGHR was significantly higher than that of cells transfected with the empty vector pCD2.1-ciR at 48 h (Figures 3F, 4F). Furthermore, the experimental condition was completed in chicken DF-1 and LMH cell lines, and similar results were displayed (Supplementary Figures 2, 3).

### CircGHR Might Promote Cell Proliferation *via* Regulating GHR and GHBP

**FIGURE 5:**
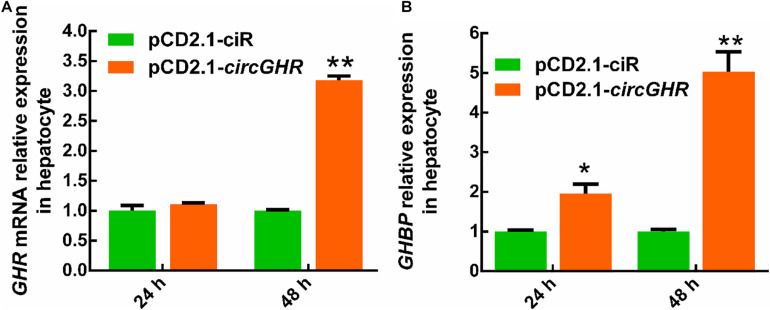
The expression profile of *GHR* gene linear transcripts after circGHR overexpression in hepatocytes. **(A,B)** The expression profile of *GHR* mRNA and *GHBP* after circGHR overexpression in hepatocytes. Fold change was relative to the expression of the cells transfected with empty vector pCD2.1-ciR at the corresponding time. All data are representative of three independent experiments and are shown as the mean ± SEM. *P < 0.05 and **P < 0.01.

**FIGURE 6:**
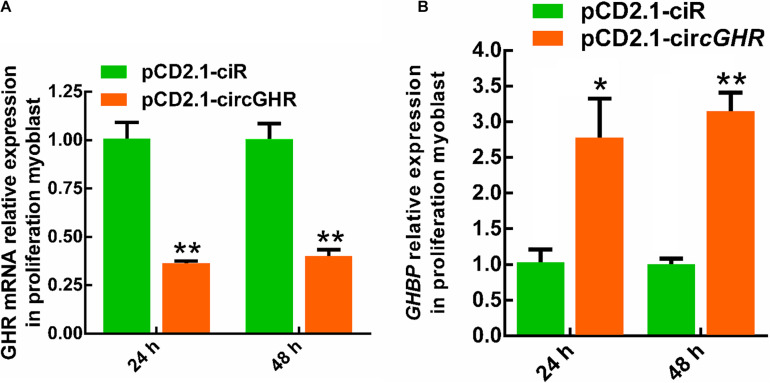
The expression profile of *GHR* gene linear transcripts after circGHR overexpression in proliferation phase myoblast. **(A,B)** The expression profile of *GHR* mRNA and *GHBP* after circGHR overexpression in myoblast during the proliferation phase. Fold change was relative to the expression of the cells transfected with empty vector pCD2.1-ciR at the corresponding time. All data are representative of three independent experiments and are shown as the mean ± SEM. *P < 0.05 and **P < 0.01.

To explore the potential mechanism of circGHR in promoting cell proliferation, the expression of *GHR* and *GHBP*, transcribed from the *GHR* gene, was detected after circGHR overexpression in the four types of cells. Various results were shown. Chicken *GHR* increased in hepatocytes (Figure 5A) and DF-1 (Supplementary Figure 4A) but decreased in myoblast (Figure 6A) and LMH cell (Supplementary Figure 5A), while *GHBP* significantly increased in all the cells (Figures 5B, 6B and Supplementary Figure 5B), except in the DF-1 cells (Supplementary Figure 4B). The proofs hinted that circGHR might affect cell proliferation by regulating *GHR* and *GHBP* production in different mechanisms that existed in different cells.

**FIGURE 7:**
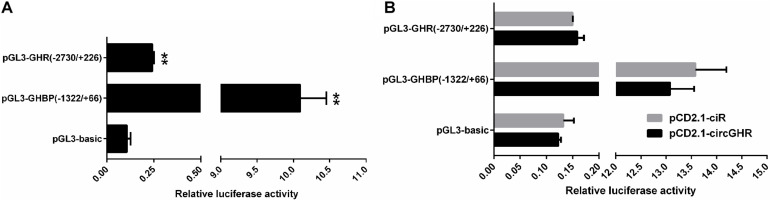
Effects of circGHR overexpression on the promoter activities of *GHR* and *GHBP* transcripts. **(A)** Effects of the promoter activities of the *GHR* and *GHBP* transcripts in DF-1 cells. **(B)** Effects of circGHR on the promoter activities of *GHR* and *GHBP* transcripts in DF-1 cells. Fold change was relative to the expression of the cells transfected with empty vector pCD2.1-ciR. All data are representative of three independent experiments and are shown as the mean ± SEM. *P < 0.05 and **P < 0.01.

To evaluate whether circGHR regulates GHR and GHBP expression, we constructed the *GHR* and *GHBP* promoter-reporter plasmid pGL3-GHR(−2,730/ +226) and pGL3-GHBP(−1,322/ +66), respectively. As expected, both plasmids’ luciferase activity was 2.28-fold and 94.74-fold higher than that of the pGL3-basic vector (*P* < 0.05, Figure 7A), suggesting that the −2,730/ +226 *GHR* and −1,322/ +66 *GHBP* had promoter activity. Then, we performed co-transfection and reporter gene assay to test whether circGHR regulates *GHR* and *GHBP* promoter activity. The reporter gene assay showed that the luciferase activity of pGL3-GHBP(−1,322/ +66) and pGL3-GHR(−2,730/ +226) was not significantly changed after circGHR overexpression (Figure 7B).

Various conserved transcription factor binding sites were predicted in the sequences of circGHR and the promoter regions of *GHR* and *GHBP* transcripts (Supplementary Table 2), like aristaless-like homeobox 3 (ALX3), AT-rich interaction domain 3A (Arid3a), nuclear factor erythroid 2 like 1 (NFE2L1), and lin-54 DREAM MuvB core complex component (Lin54) (more detailed information listed in Supplementary Table 2). The Lin54 is especially an essential regulator of cell cycle genes by binding to the promoters of G2/M genes whose products are required for mitosis, and participates in their cell cycle-dependent activation (Schmit et al., 2007).

## Discussion

Until now, several chicken circRNAs have been found, involved in liver and muscle development (Li et al., 2019). In a previous study, we identified a circRNA transcribed from the *GHR* gene and strongly resisted the exonuclease RNase R (Zhang et al., 2019). Also, reverse transcription showed to be less effective in circGHR using oligo(dT)_18_ primers than random primers. These experiments not only confirmed that circGHR was a circRNA but also laid the foundation for the following experiments. All the RNA should be transcribed with oligo(dT)_18_ and random mixture primers before qRT-PCR.

Myofiber ontogenesis begins very early during embryonic life, with the total number of fibers fixed during fetal life (Xu et al., 2019). In this study, the circGHR expression increased in the liver and decreased in the breast and thigh muscle with chicken development, suggesting that circGHR plays an essential role in chicken embryonic muscle development. In a previous study, we found similar expression trends of *GHR* mRNA in the liver (Wang et al., 2019) and myoblast (Zhang et al., 2016). The metabolic function is gradually improved during chicken liver development, impacting myoblast proliferation in the embryo and early postnatal stage (Welle et al., 2007; Zhou et al., 2013). The positive correlations between circGHR and *GHR* mRNA hinted that circGHR was closely involved in chicken development.

Animal growth and development were affected by the GH–GHR–IGF1 pathway, in which cell proliferation is a meaningful and influential process (Luo et al., 2018). We analyzed the effects of circGHR on cell proliferation in four types of cells and found that circGHR not only can promote cell proliferation but also can increase marker gene expression, indicating that circGHR was closely related to chicken development. Many researchers concluded that circRNAs are involved in animal development *via* regulating their parent’s gene expression (Li et al., 2015; Qin et al., 2018), especially for the circRNAs rich in the nucleus. Chicken circGHR was richer in the nucleus than in the cytoplasm in both hepatocytes and myoblast. Thus, we inferred that circGHR might promote cell proliferation *via* regulating *GHR* mRNA and *GHBP* expression. However, dual-luciferase reporter gene assay revealed that the luciferase activity of pGL3-GHBP(−1,322/ +66) and pGL3-GHR(−2,730/ +226) was not significantly changed after circGHR overexpression (Figure 7B). Hence, the complex concrete regulation mechanisms need further investigation.

CircRNAs regulate gene transcription at the initiation and elongation steps as well as during post-transcription. Li et al. reported that circEIF3J and circPAIP2 performed a scaffold for combining with U1 snRNPs and polymerase II in increasing parental gene *EIF3J* and *PAIP2* mRNA expression, respectively (Li et al., 2015). Additionally, ci-ankrd5, ci-sirt7, and ci-mcm5, transcribed from intron sequences, regulated homologous linear RNA transcript expression by associating with phosphorylated polymerase II, which is pivotal to the transcription elongation process (Zhang et al., 2013). Furthermore, circMbl contains several MBL protein-binding sites and, thus, can act as an RBP sponge by binding and sequestering the MBL protein, lowering its free cellular concentration so that is can no longer produce circMbl but manufacture mbl linear transcripts, suggesting that circMbl regulates circMbl and *Mbl* linear transcript expression in posttranscriptional patterns (Ashwal-Fluss et al., 2014). Some circRNAs sequester the translation start site on their linear transcripts to monitor their protein expression level, like “mRNA trapping” competing with linear transcript expression (Chao et al., 1998).

The sequence of circGHR contained 5′ UTR of the *GHR* gene, reminding us that both 5′ UTR and the intron cannot be translated and that 5′ UTR can be considered as the intron original circRNA. Coincidentally, *GHR* mRNA and *GHBP* are transcribed from different transcription initiation sites and polyadenylation sites of the *GHR* gene, thus forming two different alternative splicings during mRNA maturation. The expression of *GHR* and *GHBP* transcripts was not always increased in circGHR, which is overexpressed among different cells, and the effects and mechanisms were partly variant in different cells, even though false-positive results may exist. Therefore, we hypothesized that circGHR is involved in the formation of two transcripts and regulated their expressions like circEIF3J and circPAIP2; however, this requires further research.

Abundance indications describing circRNAs interacting with transcription factors existed. Circ-CTNNB1 bound EDAD-box polypeptide 3 (DDX3) to facilitate its physical interaction with the transcription factor Yin Yang 1 (YY1), resulting in the transactivation of YY1 and transcriptional alternation of downstream genes (Yang et al., 2019). circ-DONSON recruits the NURF complex to the SOX4 promoter and initiates its transcription (Ding et al., 2019). Interestingly, numerous conserved transcription factor binding sites were predicted in the sequences of circGHR and the promoter regions of *GHR* and *GHBP* transcripts, like ALX3, Arid3a, NFE2L1, and Lin54 (Supplementary Table 2). Especially, lin54 is an essential regulator of cell cycle genes, which may be the reason to explain that circGHR promoted cell proliferation in this study (Schmit et al., 2007, 2010).

## Conclusion

In summary, we proved that circGHR positively regulated chicken cell proliferation. CircGHR was a closed-loop structure molecule and richer in the nucleus in hepatocytes and myoblast. Quantitative real-time PCR exhibited that circGHR was increased in the liver from E13 to 7W but decreased in thigh and breast muscle. Further analysis showed that circGHR overexpression promoted cell proliferation and biomarker gene expression, as well as regulated *GHR* and *GHBP* expression in various types. Additionally, circGHR had no significant effects on the promoter activity of *GHR* and *GHBP* in the DF-1 cell line.

## Data Availability Statement

The datasets presented in this study can be found in online repositories. The names of the repository/repositories and accession number(s) can be found in the article/Supplementary Material.

## Ethics Statement

The animal study was reviewed and approved by Animal Care Committee of Guangdong Ocean University. Written informed consent was obtained from the owners for the participation of their animals in this study.

## Author Contributions

HX and LZ participated in the study design and coordination and wrote the first draft of the manuscript. HX, QL, JZ, PA-A, TL, and ZW carried out the experiments and statistical analysis. SL was involved in data interpretation and revision. LA, ZZ, and LZ participated in writing the final versions of the manuscript. All authors read and approved the final manuscript.

## Conflict of Interest

The authors declare that the research was conducted in the absence of any commercial or financial relationships that could be construed as a potential conflict of interest.

## Footnotes

1 http://jaspar.genereg.net/

## Supplementary Material

Supplementary Figure 1 The experiment summary technical pipeline about this research paper.

Supplementary Figure 2 The cell status and the expression profile of the proliferation gene after circGHR overexpression in DF-1 cell. **(A)** The status of DF-1 cell after transfection vectors 48 h. **(B–D,E)** The expression profile of circGHR, *PCNA*, *CCND1*, and *CDK2* after circGHR overexpression in DF-1 cell. Fold change was relative to the expression of the cells transfected with empty vector pCD2.1-ciR at the corresponding time. **(F)** DF-1 cell growth curves following the transfection of pCD2.1-ciR and pCD2.1-circGHR. Fold change was relative to the initial value. All data are representative of three independent experiments and are shown as the mean ± SEM. ^∗^*P* < 0.05 and ^∗∗^*P* < 0.01.

Supplementary Figure 3 The cell status and the expression profile of the proliferation gene after circGHR overexpression in LMH cell. **(A)** The status of LMH cell after transfection vectors 48 h. **(B–D,E)** The expression profile of circGHR, *PCNA*, *CCND1*, and *CDK2* after circGHR overexpression in LMH cell. Fold change was relative to the expression of the cells transfected with empty vector pCD2.1-ciR at the corresponding time. **(F)** LMH cell growth curves following the transfection of pCD2.1-ciR and pCD2.1-circGHR. Fold change was relative to the initial value. All data are representative of three independent experiments and are shown as the mean ± SEM. ^∗^*P* < 0.05 and ^∗∗^*P* < 0.01.

Supplementary Figure 4 The expression profile of *GHR* gene linear transcripts after circGHR overexpression in DF-1 cell. **(A,B)** The expression profile of *GHR* mRNA and *GHBP* after circGHR overexpression in DF-1 cell. Fold change was relative to the expression of the cells transfected with empty vector pCD2.1-ciR at the corresponding time. All data are representative of three independent experiments and are shown as the mean ± SEM. ^∗^*P* < 0.05 and ^∗∗^*P* < 0.01.

Supplementary Figure 5 The expression profile of GHR gene linear transcripts after circGHR overexpression in LMH cell. **(A,B)** The expression profile of *GHR* mRNA and *GHBP* after circGHR overexpression in LMH cell. Fold change was relative to the expression of the cells transfected with empty vector pCD2.1-ciR at the corresponding time. All data are representative of three independent experiments and are shown as the mean ± SEM. ^∗^*P* < 0.05 and ^∗∗^*P* < 0.01.

Supplementary Table 1 Original data.

Supplementary Table 2 The conserved transcription factors in circGHR and the promoter regions of *GHR* and *GHBP* transcripts.
