# Supplementary figures and images for: Effects of Circular RNA of Chicken Growth Hormone Receptor Gene on Cell Proliferation
Source: Front Genet. 2021 Feb 11;12:598575. doi: 10.3389/fgene.2021.598575 (PMC7905176; doi:10.3389/fgene.2021.598575)

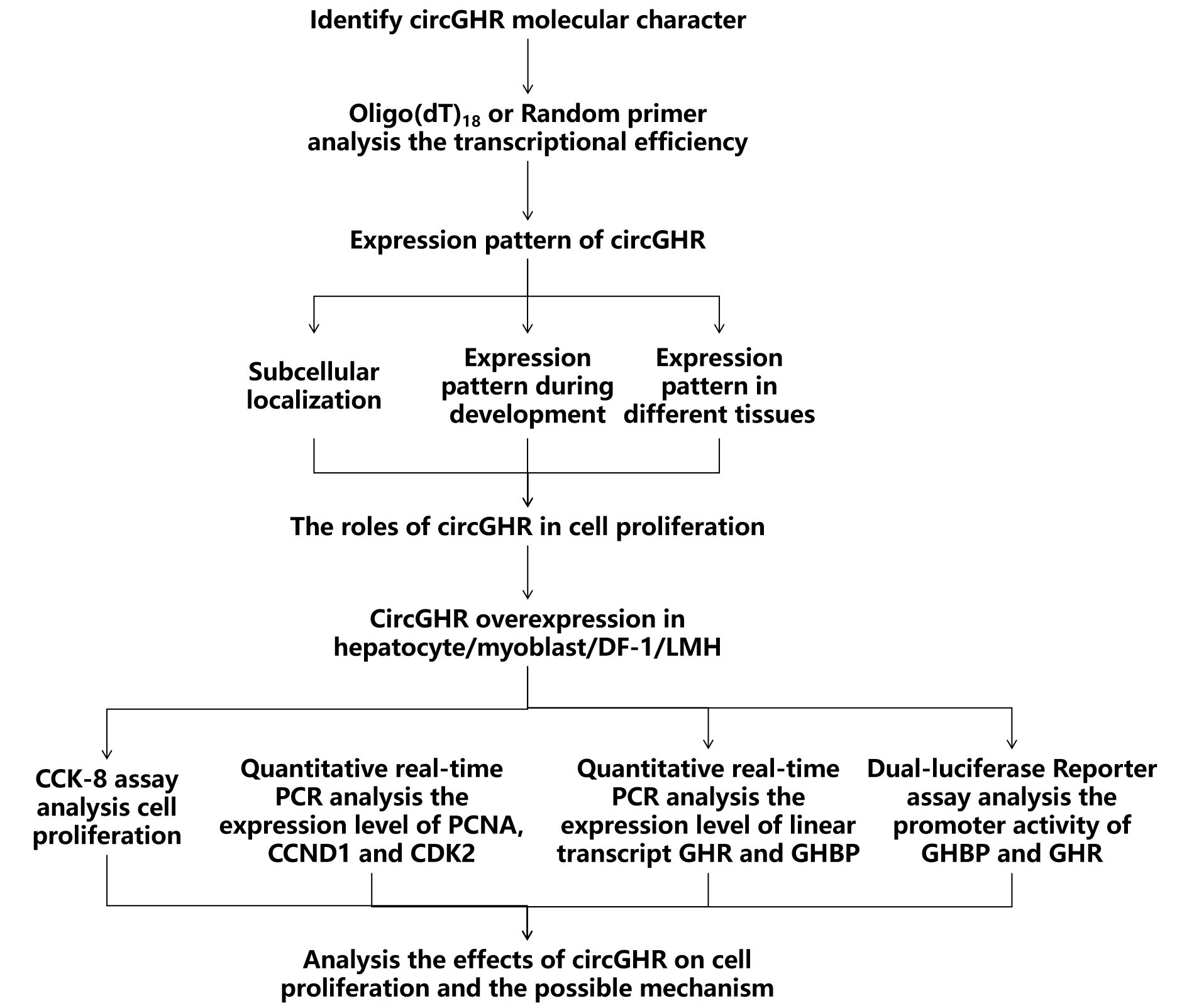

Supplement: Supplementary Figure 1 — The experiment summary technical pipeline about this research paper. [file Image_1.TIF]

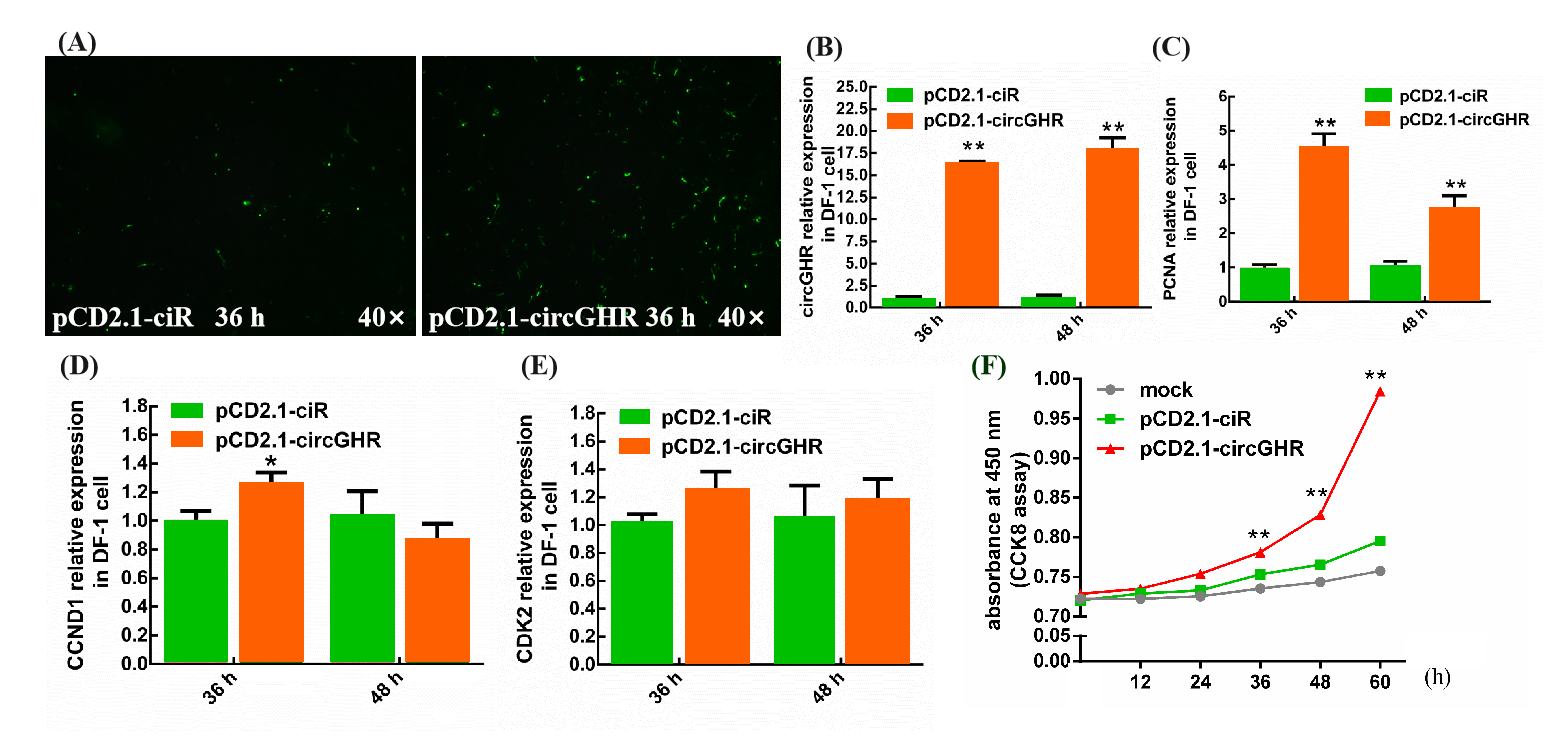

Supplement: Supplementary Figure 2 — The cell status and the expression profile of the proliferation gene after circGHR overexpression in DF-1 cell. (A) The status of DF-1 cell after transfection vectors 48 h. (B–D,E) The expression profile of circGHR, PCNA, CCND1, and CDK2 after circGHR overexpression in DF-1 cell. Fold change was relative to the expression of the cells transfected with empty vector pCD2.1-ciR at the corresponding time. (F) DF-1 cell growth curves following the transfection of pCD2.1-ciR and pCD2.1-circGHR. Fold change was relative to the initial value. All data are representative of three independent experiments and are shown as the mean ± SEM. ∗P < 0.05 and ∗∗P < 0.01. [file Image_2.TIF]

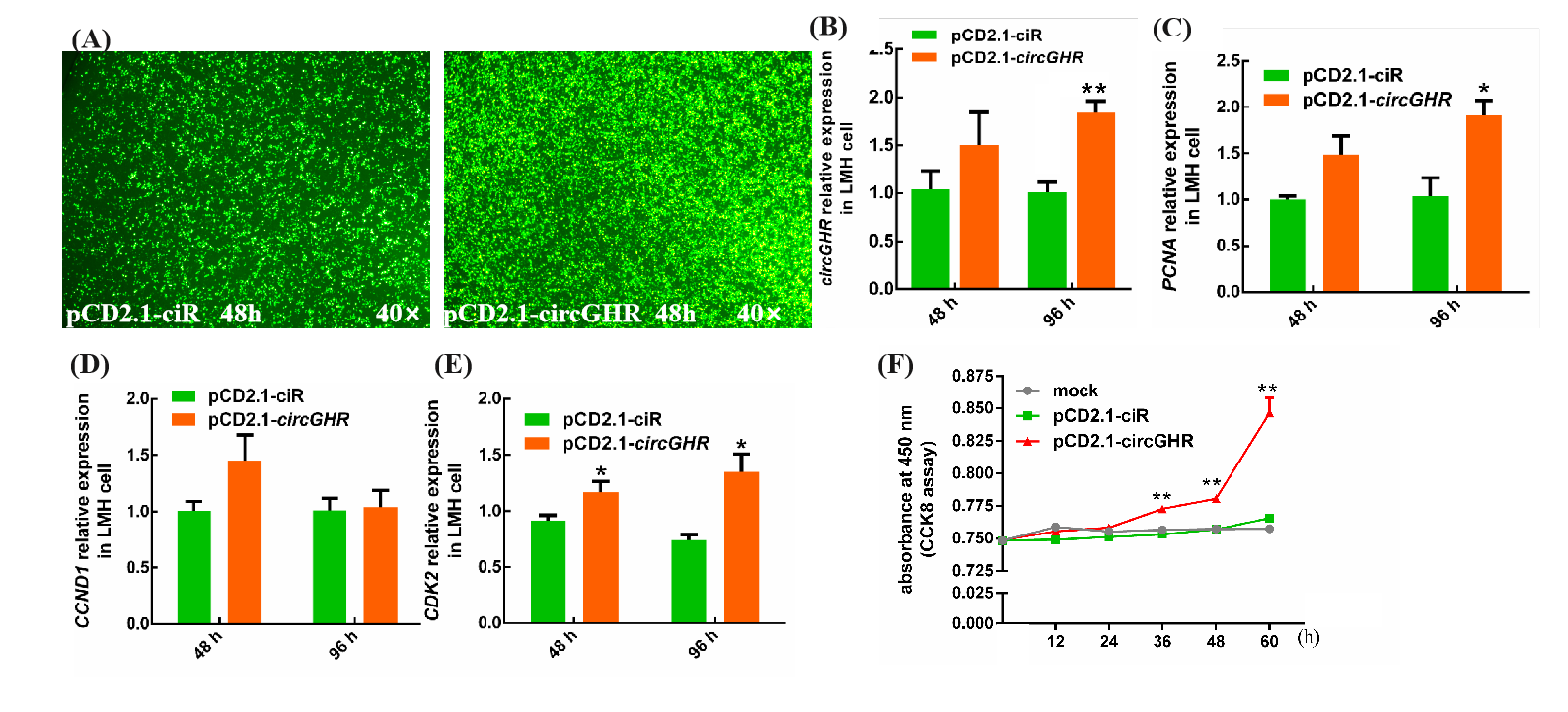

Supplement: Supplementary Figure 3 — The cell status and the expression profile of the proliferation gene after circGHR overexpression in LMH cell. (A) The status of LMH cell after transfection vectors 48 h. (B–D,E) The expression profile of circGHR, PCNA, CCND1, and CDK2 after circGHR overexpression in LMH cell. Fold change was relative to the expression of the cells transfected with empty vector pCD2.1-ciR at the corresponding time. (F) LMH cell growth curves following the transfection of pCD2.1-ciR and pCD2.1-circGHR. Fold change was relative to the initial value. All data are representative of three independent experiments and are shown as the mean ± SEM. ∗P < 0.05 and ∗∗P < 0.01. [file Image_3.TIF]

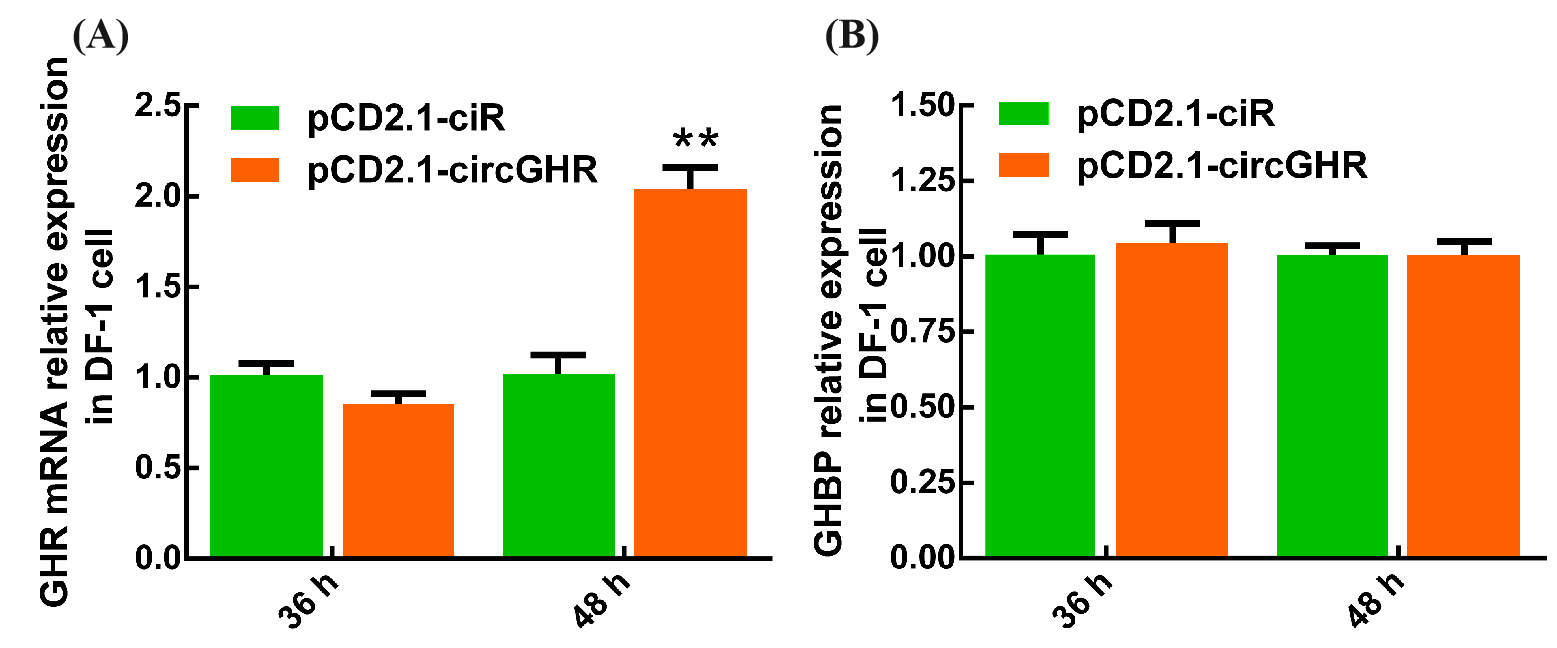

Supplement: Supplementary Figure 4 — The expression profile of GHR gene linear transcripts after circGHR overexpression in DF-1 cell. (A,B) The expression profile of GHR mRNA and GHBP after circGHR overexpression in DF-1 cell. Fold change was relative to the expression of the cells transfected with empty vector pCD2.1-ciR at the corresponding time. All data are representative of three independent experiments and are shown as the mean ± SEM. ∗P < 0.05 and ∗∗P < 0.01. [file Image_4.TIF]

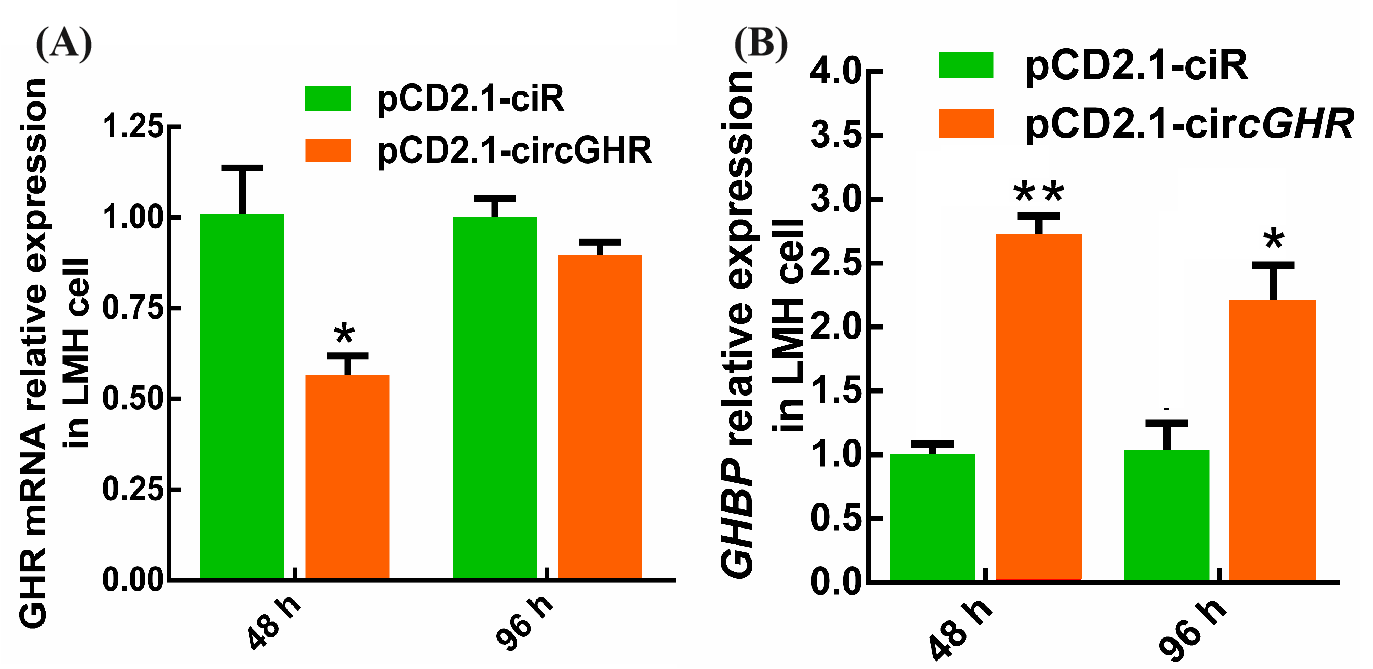

Supplement: Supplementary Figure 5 — The expression profile of GHR gene linear transcripts after circGHR overexpression in LMH cell. (A,B) The expression profile of GHR mRNA and GHBP after circGHR overexpression in LMH cell. Fold change was relative to the expression of the cells transfected with empty vector pCD2.1-ciR at the corresponding time. All data are representative of three independent experiments and are shown as the mean ± SEM. ∗P < 0.05 and ∗∗P < 0.01. [file Image_5.TIF]
